# Improving Knowledge, Attitude and Perception of ECT Amongst CWPT Healthcare Workers

**DOI:** 10.1192/bjo.2025.10419

**Published:** 2025-06-20

**Authors:** Onyedikachi Onyeaso, Omolayo Apantaku, Regina Ugwu, Sashriya Singh, Samina Azeem

**Affiliations:** Coventry and Warwickshire Partnership NHS Trust, Coventry, United Kingdom

## Abstract

**Aims:** Electroconvulsive Therapy (ECT) remains a misunderstood and underutilized treatment option in psychiatric care, often due to misconceptions and biases among healthcare professionals. This Quality project aimed to identify the perceptions, attitudes, and biases toward ECT among ward staff at Coventry and Warwickshire Partnership Trust (CWPT) and to improve their knowledge and attitudes through targeted educational interventions. The project sought to address the lack of access to accurate information about ECT, which has led to its perception as an inhumane treatment, overshadowing its therapeutic benefits.

**Methods:** The project involved a pre-intervention survey to assess baseline knowledge, perceptions, and attitudes toward ECT among 32 CWPT ward staff. Following this, a teaching session was organized to disseminate accurate information about ECT, its applications, and its benefits. Post-teaching questionnaires were administered to evaluate the impact of the intervention. The Plan-Do-Study-Act (PDSA) cycle was used to guide the intervention and measure outcomes.

**Results:** The intervention led to significant improvements in staff knowledge, perceptions, and attitudes toward ECT. Key findings included a 42.6% increase in positive attitudes toward ECT, a 22.6% improvement in knowledge, and a 12.9% increase in confidence levels when discussing ECT with patients. Staff reported higher awareness of ECTs applications and effectiveness. Willingness to recommend ECT as a treatment option also increased by 6.5%. These results highlight the importance of targeted education in addressing misconceptions and biases among healthcare professionals.

**Conclusion:** This project successfully improved the knowledge, perceptions, and attitudes of CWPT ward staff toward ECT. The findings underscore the need for ongoing education and access to accurate information about ECT within psychiatric services. Recommendations include incorporating ECT teaching into e-learning resources for staff and continuing to provide opportunities for staff to observe ECT procedures. These efforts can help ensure that ECT is recognized as a valuable and humane treatment option in psychiatric care.

